# Recent Developments in the Assessment of Nutrition Knowledge in Athletes

**DOI:** 10.1007/s13668-022-00397-1

**Published:** 2022-02-16

**Authors:** Ryan Tam, Janelle A. Gifford, Kathryn L. Beck

**Affiliations:** 1 School of Behavioural and Health Sciences, Faculty of Health Sciences, Australian Catholic University, 22 Main St, Blacktown, NSW 2148 Australia; 2 Sydney School of Health Sciences, Faculty of Medicine and Health, The University of Sydney, Susan Wakil Health Building, Camperdown NSW 2006, Sydney, Australia; 3 School of Sport, Exercise and Nutrition, College of Health, Massey University, Palmerston North, New Zealand

**Keywords:** Sports nutrition, Nutrition knowledge, Knowledge questionnaire, Athlete knowledge, Validity, Reliability, Questionnaire development

## Abstract

***Purpose of Review*:**

Adequate nutrition knowledge may influence dietary behaviour, and the performance and health of athletes. Assessment of the nutrition knowledge of athletes can inform practice and provide a quantitative way to evaluate education interventions. This article aims to review nutrition knowledge questionnaires published in the last 5 years to identify advances, possible improvements in questionnaire development and design, and challenges that remain.

***Recent Findings*:**

Twelve new or modified questionnaires were identified. All had undergone validity and reliability testing. Advancements included quantitative measures of content validity and Rasch analysis. Online questionnaires were common, with at least seven using this format. Advances included use of images (*n* = 2), automated scored feedback (*n* = 1), and use of applied questions.

***Summary*:**

While advancements have been made in validation and reliability testing and electronic delivery, new questionnaires would benefit from interactive and attractive features including images, provision of electronic feedback, and applied questions.

## Introduction

In sport, nutrition and performance are inextricably linked [1]. Dietary intake supplies the necessary energy and nutrients to meet training demands; for tissues to adapt, repair, and grow; and to promote immune function. During competition, emphasis is placed on the use of nutrition strategies to delay or prevent performance decrements related to fatigue or dehydration [2]. The use of planned nutrition strategies has been shown to improve performance when compared to ad libitum strategies [3, 4].

Hunger, taste, cost, and convenience are important determinants of food selection; however, an athlete’s choices are more complex due to a need to concurrently consider performance expectations, effects on physique, stage of training, and proximity to competition [5]. Given the range of factors that impact on food selection, knowledge of nutrition is necessary to help inform decisions around food choice. Without knowledge of the benefits of certain foods and nutrients, or the potential individual benefits from consuming these, athletes are not able to make considered decisions for their inclusion within their diet [6].

Nutrition knowledge and dietary quality have been shown to be positively associated in both the general population and in athletes [7, 8], although this relationship has been difficult to quantify due to the limited availability of validated instruments for this purpose [9]. Several recent studies have reported improvements in nutrition knowledge and dietary intake after educational interventions in a variety of sports and athlete types [10–13] which further suggests that the improvement of nutrition knowledge is linked to dietary intake. However, to give meaning to these results, the accurate assessment and quantification of knowledge level are necessary.

Sports nutritionists (SN) and other practitioners such as coaches and trainers working with athletes often play the role of educators. Nutrition knowledge assessment provides a means to measure progress over time, quantifies the effectiveness of education provided [14, 15], and facilitates the appropriate pitching of nutrition education especially in group settings. Sports nutritionists who are often working with large numbers of athletes in teams or at institutions [16] may benefit from using knowledge assessment tools to help prioritise athletes that require more urgent intervention.

Several reviews investigating nutrition knowledge in athletes have continued to identify the ongoing need for valid and reliable nutrition knowledge assessment tools for athletes [7, 17]. A review in 2016 found tools used in the measurement of nutrition knowledge in athletes were inadequately validated, making it difficult to ascertain the nutrition knowledge of athletes [18]. This manuscript aims to summarise nutrition knowledge questionnaires that have been published in the last 5 years to identify advances, where improvements in questionnaire development can still be made, and challenges that remain in this research space.

## Search Strategy and Results

A search across two databases (PubMed and Web of Science) was conducted using terms including (“nutrition knowledge” OR “nutrition assessment” OR “knowledge assessment”) AND (“athlet*” OR “sport*”) AND (“questionnaire” OR “tool” OR “instrument”). Results were limited to the last 5 years (2016 onwards). To be included, articles had to describe the development of a nutrition knowledge questionnaire for any athlete population from any country and be published in English. Abstracts, theses, and reviews were excluded. Where possible, a copy of the questionnaire was retrieved.

Twelve questionnaires were identified through the search. Information describing each questionnaire including population, athlete type, delivery platform, assessment areas, questionnaire length, type of questions, scoring information, and validity and reliability testing were doubly extracted by two authors (R.T, K.B). The Nutrition for Sports Knowledge Questionnaire (NSKQ) [19] and its abridged version (A-NSKQ) by Trakman et al. were considered as separate questionnaires for the purpose of this review [20].

## Results

A summary of the twelve questionnaires included in this review is provided in Table 1. Questionnaires have been developed by researchers from around the world (Australia, *n* = 3 [19–21]; UK, *n* = 2 [22, 23]; Turkey, *n* = 2 [24, 25]; Italy, *n* = 2 [26, 27]; and Spain [28•], Finland [29], and the USA, *n* = 1 [30]) with the majority targeting all athlete types (*n* = 8). The remaining questionnaires specifically targeted endurance [29], ultra-endurance [22], sports teams [28•], and track and field athletes [23]. One questionnaire was aimed specifically at early adolescents [27], and two were targeted at adolescents/youths as well as adults [26, 28•].

**Table 1:** Summary of sports nutrition knowledge questionnaires

**Author name, country, year**	**Questionnaire**	**Athlete group**	**Delivery mode**	**Topics**	**Number of questions and scoring, time to complete**	**Validation measures**	**Reliability measures (and how)**	**Other features**
Blennerhassett, UK, 2019 [22]	ULTRA-Q (modified, Zinn) [37]	Ultra-endurance	Online	General and sports nutrients, fluid, recovery, weight management, supplements	76 H/M/L (see Zinn) [37] 1 = correct 0 = all other responses	Reviewed by 4 external SN RDs (**content validity**) **Pilot** testing (10 SENR, 10 RD, 13 GenP, *p* < 0.001) (known groups, **construct validity**)	**Test–retest** (2 wks later) (*n* = 29): ICC = 0.75–0.95 **Internal consistency** (101 ultra-endurance athletes): = 0.63–0.87	
Calella, Italy, 2017 [26]	GeSNK (new)	Adolescent and young adults, general	Unclear	General (29 items) Macro/micronutrients in various foods, practical food choices, awareness of diet-health associations Sports nutrition (33 items) Fluid replacement, supplement intake, food and timing choices in recovery meals	62 (max score 97) T/F/DK H/L/absent/DK 1 = correct 0 = incorrect/DK	Expert review by 2 paediatricians, 2 dietitians, 2 psychologists (**content validity**) **Pilot** testing - 250 high school students **Item difficulty analysis** **Item discrimination index** 2 other NK questionnaires administered, PCC = 0.456 and 0.470 (**concurrent validity**) 49 dietetic students/dietitians vs 83 sports scientists vs movement science students vs 57 economists/economic students, *p* < 0.001 (known groups - **construct validity**)	**Test–retest** (2 wks later) (*n* = 137): PCC = 0.85 **Internal reliability** (202 high school students, active and inactive): Cronbach’s *α* = 0.86	Released in Italian Does not cover alcohol Low, medium, and high profile of knowledge based on tertiles
Furber, UK, 2017 [23]	NKQA (new)	Track and field	Unclear	Macronutrient and general, hydration and sport specific	62 H/L/not sure A/D/not sure MC Y/N/unsure 15 min, 20 s	Reviewed by experts (2 graduate SN, 2 nutritionists, 1 physiologist) (**content validity**) 53 SN and dietitians vs 48 professionals and postgraduate students with no nutrition, *p* < 0.05 (known groups, **construct validity**) **Pilot** - 58 athletes tested duration to complete	**Test–retest** (3 wks later): PCC = 0.87–0.98 **Internal consistency:** Cronbach’s *α* = 0.78–0.92	
Heikkila, Finland, 2017 [29]	No name (new)	Endurance (including coaches)	Online	General nutrition, athletic performance, recovery, fluids, supplements, weight control	79 T/F	6 SN experts reviewed (**content, face validity**) Pre-pilot testing (6 athletes and researchers) **Pilot** testing (16 athletes 15–20 y, 13 coaches (23–52 y) **Item difficulty index** 20 nutrition vs 22 humanities students, *p* < 0.01 (**construct validity**)	**Test–retest** (5 wks later): PCC = 0.85 **Internal consistency**: Cronbach’s *α* = 0.87	Available in English, Swedish, and Finnish
Karpinski, US, 2019 [30]	49-SNKI (new)	Adults, general	Online (Qualtrics ™)	Sports nutrition focus only (carbohydrates, protein, fat, hydration, energy balance/weight management, micronutrients)	49 T/F/DK 1 = correct − 1 = incorrect 0 = DK	Modified Delphi survey (2 rounds) with 24 sports RDNs **(face, content validity)** **Pilot** testing (31 athletes, 6 RDNs) **Item difficulty index** 202 athletes vs 56 RDNs, *p* < 0.01 (**construct validity**) Qualitative review of feedback received	**Test–retest** (2 wks later) (12 athletes, 8 RDN): PCC = 0.951 **Internal consistency:** Cronbach’s *α* = 0.843	Tested readability and reading grade level using the Gunning Fog Index and Flesch Kincaid Grade Level Index respectively
Okta, Turkey, 2021 [24]	GeSNK (modified, Callela) [26]	General	Unclear	General (25 items), sport (24 items) Refer to Calella [26]	49 T/F/DK H/L, NP/DK Refer to Calella [26]	Back translated into Turkish by 3 experts Consulting experts **(content validity)** **Item difficulty analysis** **Item discrimination index** (401 male soccer players, 10–19y)	**Internal consistency:** Cronbach’s *α* = 0.884	
Ozener, Turkey, 2021 [25]	No name (modified, Zinn) [37]	General	F2F	General and sports (nutrients, fluid, recovery, weight management, supplements)	78 Refer to Zinn 1 = correct 0 = incorrect/not sure	SNKQ back translated into Turkish 5 RDs reviewed to make foods culturally appropriate (**content validity**) **Pilot** testing (25 athletes) 85 dietetic students vs 125 athletes (*p* < 0.001) (known groups, **construct validity)**	**Test–retest** (2 weeks) (24 athletes, 18 dietetic students): ICC = 0.974 **Internal consistency**: KR-20 = 0.927	
Rosi, Italy, 2020 [27]	No name (new)	Early adolescents (12–14 y), general	Unclear	General (nutritional content of several foods, recommendations of nutrients, role of nutrients in health); Sports (relationship between nutrition and sport, e.g. pre-training snack, water consumption during activity, use of supplements)	26 T/F/DK MC including DK 1 = correct 0 = incorrect/DK	Experts (6 nutritionists, 2 SN experts) (**content validity**) 264 adolescents 12–14 y: footballers (67%) and non-athletes (33%) **Item difficulty analysis** **Item discrimination index** Nutrition lessons (43%) vs no nutrition lessons (57%), *p* < 0.001 (**construct validity**)	**Test–retest** (3 wks later) (39 adolescents): PCC = 0.977 **Internal consistency:** Cronbach’s *α* = 0.684	
Tam, Australia/NZ, 2021 [21, 31, 42•]	PEAKS-NQ (new)	General	Online (FileMaker Pro™)	General and sports (food groups, nutrients, applied sports nutrition, competition nutrition, supplements, and special concerns)	Final version - 50 items (maximum score 75) MC 1 = correct 0 = incorrect/not sure Estimated 15 min	4 focus groups with SN experts in Australia and NZ, modified Delphi process + NZ SN expert review for NZ athletes (**content validity**) 45 Australian SD (**S-CVI**) **Pilot** - 88 developing athletes from 13 sports vs 45 Australian SD, *p* < 0.001 (known-groups, **construct validity**) **Rasch analysis** - unidimensionality, DIF	**Test–retest** (at least 2 wks later) (*n* = 18 athletes): PCC = 0.77, ICC = 0.68 **Internal consistency:** Cronbach’s *α* = 0.86 **Rasch analysis** - high item and person reliability **Bland and Altman analysis** - no systematic bias	Uses images and provides feedback for participants Includes questions on applied “how to” SN, e.g. selecting foods for specific scenarios
Trakman, Australia, 2017 [19]	NSKQ (new)	General	Online	Weight management, macronutrients, micronutrients, sports nutrition (hydration, the pre-competition meal, nutrition during exercise, recovery nutrition), supplementation, alcohol	89 A/D/NS MC Effective/Not effective/Not sure 1 = correct 25 min	Items developed by 4 academics, reviewed by 3 academics **CVI** with 3 sports dietitians Feedback and think out loud protocol from 8 student athletes (**face validity**) 188 athletes **Item difficulty index** **Item discrimination index** **Distractor utility** **Rasch analysis** (fit residuals, DIF, disordered thresholds; 3 of 6 subsections met the Rasch model) 181 athletes Nutrition education (52%) vs no nutrition education (48%), *p* < 0.01, (known-groups, **construct validity**)	**Test–retest** (10 d to 2 wks later) (*n* = 28)-PCC = 0.92 **Internal consistency:** KR-20 = 0.88	Includes pictures to reduce respondent fatigue (details not given)
Trakman, Australia, 2018 [20]	A-NSKQ (modified, Trakman) [19]	General	Online (Qualtrics™)	General (17 items), Sports (20 items) Weight management, macronutrients, micronutrients, supplementation, sports nutrition, alcohol	37 See Trakman [19] Missing responses = incorrect 12 min	**Rasch analysis** (Item fit residuals, item characteristic curves, category probability curves, DIF; general and sports sections fitted the model) 181 athletes Nutrition education vs no nutrition education, *p* < 0.001 (**construct validity**) Individuals’ score (%) on NSKQ and proxy score on A-NSKQ - correlation = 0.9	**Test–retest** (10 d to 2 wks later): PCC = 0.7 **Rasch analysis** (general and sports sections - unidimensional, Person separation index = 0.8)	177 netballers and Australian Rules footballers-Low response rates (7%) but high completion rates (85%)
Vazquez-Espino, Spain, 2020 [28•]	NUKYA (new)	Youth and adults (sports teams)	Online	Macronutrients, micronutrients, hydration, food intake periodicity	59 MC including DK/not sure 1 = correct Negative scoring system Incorrect = 1/*n* - 1 (*n* is number of options in test item) 12 min	Items generated by researchers and sports club medical experts Reviewed by 12 international experts with multiple career backgrounds in sport I-CVI, S-CVI (**face validity, content validity**) **Item difficulty index** **Item discrimination index** **Rasch** rating scale model (extended Rasch modelling) **Pilot** (footballers 12–15 y) 37 athletes vs 49 nutrition university students vs 39 non-nutrition university students vs 93 high school students, *p* < 0.00001 (**discriminant/construct validity**)	**Test–retest** (2–4 wks later) (*n* = 173): PCC = 0.895 Split-half reliability: *r* = 0.801 and 0.839 **Internal consistency:** Cronbach’s *α* = 0.849 **Rasch global separation reliability** = 0.861	Available in Spanish and English Does not cover supplement intake, weight control, alcohol consumption

*A* agree, *A-NSKQ* Abridged-Nutrition for Sport Knowledge Questionnaire, *CVI* Content Validity Index, *I-CVI* Item-Content Validity Index, *S-CVI* Scale-Content Validity Index, *DIF* differential item functioning, *D* disagree, *DK* don’t know, *F* false, *F2F* face to face, *GeSNK* General and Sports Nutrition Knowledge Questionnaire, *GenP* general population, *H* high, *ICC* intra-class correlation, *KR* Kuder-Richardson, *L* low, *M* medium, *MC* multi-choice, *N* no, *NP* not present, *NKQA* Nutrition Knowledge Questionnaire for Athletes, *NS* not sure, *NSKQ* Nutrition for Sport Knowledge Questionnaire, *NUKYA* Nutrition Knowledge for Young and Adult Athletes, *NZ* New Zealand, *PCC* Pearson’s correlation coefficient, *PEAKS-NQ* Platform to Evaluate Athlete Knowledge of Sports Nutrition Questionnaire, *RD* registered dietitian, *RDN* registered dietitian nutritionists, *SENR* sport and exercise nutrition register, *SD* sports dietitian, *SN* sports nutrition, *SNKI* Sports Nutrition Knowledge Instrument, *T* true, *UK* United Kingdom, *US* United States of America, *Y* yes

Most of the questionnaires were newly developed (*n* = 8) with the remainder being modifications of previously validated instruments. One questionnaire was modified as a shortened version of the original [20], two were adapted for Turkish audiences [24, 25], and the last was altered to suit ultra-endurance athletes [22].

The distribution of questionnaires was described as online, electronic, or an email link for seven studies [19–22, 28•, 29, 30]; however, usually no further details were provided. Qualtrics™ (Provo, UT, USA) and FileMaker Pro™ (Cupertino, CA, USA) were used as the platform for three questionnaires [19, 21, 30].

### Validity and Reliability Measures of Questionnaires

In author-developed questionnaires (*n* = 8), an expert panel was always used to establish face validity (does the questionnaire appear to measure what it claims to?) and/or content validity (is the questionnaire representative of the domain being assessed?) [19, 21, 23, 26, 27, 28•, 29, 30]. The PEAKS-NQ differed by conducting focus groups with SN from elite sporting institutions to inform the generation of items, resulting in high consensus and no deletions during refinement via a modified Delphi process [31]. These experts also identified other desirable features in nutrition knowledge questionnaires. These included a modular approach to allow the selection of the most relevant assessment areas, a rotating question bank, visual appeal (e.g. images), immediate feedback, and electronic deployment.

While the use of an expert panel is deemed appropriate for establishing content validity [32, 33••], expert groups used in the literature are diverse, including SN with differing levels of experience [23], experts from multiple career backgrounds [28•], psychologists, and paediatricians [26]. These experts’ training in sports nutrition were not described. In most questionnaires, content validity was established qualitatively; however, three recent instruments also included quantitative measures [19, 21, 28•]. The content validity index (CVI) was used differently in each study, with the NSKQ using an expert panel of nutritionists to rate each item [19]. The NUKYA [28•] assessed the CVI both as individual items (I-CVI) and as whole scale (S-CVI), whereas each section of the PEAKS-NQ [21] was rated by SNs then averaged to establish an overall S-CVI.

Construct validity, which refers to how well a questionnaire measures the variable that it intends to [34, 35], was most often supported by using known-groups validity, also referred to as discriminative validity or validation by extreme groups [32, 36]. Most questionnaires recruited a “high knowledge” group such as practicing nutritionists or nutrition students and a comparison group who were expected to have a lower level of nutrition knowledge such as athletes or university students with no nutrition training. Demonstrating a questionnaire’s ability to distinguish between differing levels of knowledge supports construct validity [32, 33••]. All questionnaires utilised this method except Okta et al. [24], who translated and modified a questionnaire which had been validated using this method [37]. The A-NSKQ did not reconduct known-groups testing after its initial development as it was developed from previously collected data [20].

Most questionnaires (*n* = 11) used a “test–retest” method to establish the reliability of questionnaires [19–23, 25–27, 28•, 29, 30]. The time between administrations ranged between 10 days [19] and 5 weeks [29]. Cronbach’s *α* was most often used to measure the internal consistency of the instrument [21, 23, 24, 26, 27, 28•, 29, 30], followed by the Kuder-Richardson 20 [19, 24, 25].

Rasch analysis was used to support the psychometric properties of four instruments [19–21, 28•]. However, the use of Rasch analysis and the subsequent statistics reported were inconsistent between studies. Rasch analysis has strong properties suited to questionnaire development including the evaluation of construct validity, refinement of test items by identifying non-discriminative items, the identification of questions not performing as expected, and assessment of reliability [38, 39, 40•].

Other methods of refining questionnaires included the use of a difficulty index to identify items that were too easy or hard, and or a discrimination index to assess how well items distinguish between high and low performing respondents [19, 24, 26, 27, 28•, 29, 30]. A “think out loud” protocol was used in one study to collect qualitative feedback from student athletes on the questionnaire [19]. Readability was assessed in one study using the Gunning Fog Index and the Flesch Kincaid Grade Level Index [30].

All questionnaires focussed on general and sports nutrition knowledge with the exception of one which focussed on sports nutrition knowledge only [30]. Nutrients (including macronutrients and micronutrients) and fluid/hydration were assessed across all questionnaires. Other topics that were frequently assessed included recovery nutrition (*n* = 5) [22, 24–26, 29], weight management (*n* = 7) [19–22, 25, 29, 30], and supplements (*n* = 9) [19–22, 24–27, 29]. Few commented on question type and the knowledge type tested, e.g. factual (declarative) or procedural (“how-to”) [41]. Procedural knowledge has been identified as important in demonstrating understanding of nutritional concepts [31].

The number of questions varied (range 26–89, mean 59 ± 18 items). The true number of items could be greater as each question may contain multiple sub-questions. For example, “The carbohydrate content of these foods is:” was reported as one question but contained six sub-questions [26]. Questionnaire completion time was reported in five studies [19–21, 23, 28•] of between 12 and 25 min. Many questionnaires predominantly used dichotomous answers (true/false, high/low, agree/disagree), with most including an “unsure” option [19, 23, 24, 26, 27, 30]. Half the instruments included multiple choice questions (MCQs) [19–21, 23, 27, 28•]. Scoring was reported in nine questionnaires [19–22, 25–27, 28•, 30] with the majority awarding + 1 for each correct and 0 for incorrect or “I don’t know” answers. Negative scoring was reported for incorrect answers in two studies [28•, 30]. The PEAKS-NQ was the only questionnaire to report automated scoring and feedback upon completion [21]. This feedback displayed scores and strengths and weaknesses in knowledge domains; these additions were reported subsequent to its validation [42•].

The use of images was mentioned in two studies [19, 21]. The NSKQ included pictures to reduce participant fatigue and the PEAKS-NQ included visual aids to improve respondent comprehension.

## Overview of Current Questionnaire Design and Suggestions for Future Developments

In the last 5 years, advances have occurred in the way athlete nutrition knowledge is assessed. The extent of validity and reliability testing and refinement of questionnaires via more diverse and sensitive techniques appear to have helped address problems identified in previous systematic reviews [7, 17, 18]. Other areas for improvement in sports nutrition questionnaire development include focusing on “how to” knowledge questions, reducing the reliance on dichotomous items, minimising ambiguous questions, incorporating electronic features and the use of consistent methodology in translating and/or modifying questionnaires.

### Testing of Validity and Reliability

A review of studies pre-2016 identified limited testing of validity and reliability; however, most newly developed questionnaires utilised at least four techniques: the use of an expert review panel for content validity, comparison of groups expected to perform differently to support construct validity, test–retest to inform questionnaire stability, and assessment of internal consistency. Tools modified from previously validated instruments conducted less testing prior to use [20, 22, 24, 25].

Most authors consulted a panel of experts to establish content validity. This could be problematic because what defines an expert is contextual. A wide range of experts were used in the validation of different questionnaires including SN with varying levels of experience, and other health professionals who may have limited nutrition training [23, 24, 28•, 29]. An alternative is the use of focus groups. Focus groups were used by Trakman and colleagues [19] to assess the clarity of the NSKQ after the item pool was generated, whereas the authors of the PEAKS-NQ used focus groups with SN to inform the initial item pool [31]. Focus groups can be useful in generating a representative item pool by giving participants an opportunity to stimulate each other’s thinking, providing more diverse perspectives that uncover researchers’ “blind spots” and can reduce researcher bias [43–45]. This method remains underutilised in this research area.

The use of quantitative methods to measure content validity has become more common. Content validity index (CVI), whether as a whole scale or by individual item, was assessed in three questionnaires, each in a slightly different manner [19, 21, 28•]. It may be too early to establish a standard practice for how CVI should be assessed; however, those developing new questionnaires should include this technique to compliment methods that use an expert panel. Considerations for how CVI can be implemented have been previously suggested [33••, 46, 47].

Another notable advance has been the inclusion of Rasch techniques in four recent questionnaires, which is a form of item response theory (IRT). Item response theory techniques consider individual question difficulty as related to person ability [44]. Evaluation of the psychometric properties of a questionnaire has traditionally been conducted via classical test theory (CTT) techniques which analyse whole scales based on total score, where the ability of the respondent is determined by overall performance [48]. Knowledge is a non-linear construct, so relying on CTT techniques can be problematic because they do not provide insight on how individuals perform on each item in relation to their ability [49]. Rasch modelling presumes that more difficult items are less likely to be answered correctly and vice versa; therefore, performance of a respondent can be predicted based on item difficulty and person ability [38]. From a questionnaire development perspective, Rasch analysis has been used to support construct validity, rank questions by difficulty and fit to identify potentially problematic or non-discriminatory items, and assess reliability by providing person and item separation reliability scores [19–21, 28•]. Reliability scores are similar to Cronbach’s *α*, but consider that data provided is non-linear which is advantageous [40•]. Different Rasch analyses were conducted by the four questionnaires, which can be attributed to the different computer programs that conduct this analysis [33••]. With its advantages over traditional techniques, developers of nutrition knowledge questionnaires should consider using a combination of IRT and CTT techniques in testing validation.

Aside from developments in validity and reliability, there remain several areas for opportunities to improve knowledge assessment in athletes. These include changes to the types of questions asked, use of images, electronic features, and modular question banks.

### A Focus on Assessing Practical (“How to”) Nutrition Knowledge

For most questionnaires, it was unclear whether they focussed on factual (declarative) or procedural (practical/ “how to”) knowledge [41]. The ability to apply nutrition principles (procedural knowledge) is impossible without factual knowledge [41]; however, a focus on facts alone (e.g. questions such as “what percentage of your diet should be made up from carbohydrate?”) [23] may limit the usefulness of information [19]. That is, ability to recall facts is unlikely to truly reflect understanding of nutrition or the ability to select foods conducive to good health and performance. Greater understanding of practical nutrition knowledge has recently been associated with higher diet quality [50]. The assessment of both factual and application-based knowledge of sports nutrition should remain a focus of future questionnaires.

### Reducing Reliance on Dichotomous Item Formats

Most questionnaires used dichotomous response formats and inclusion of a “not sure” option. There are arguments for and against the inclusion of an “unsure” option. Inclusion may decrease likelihood of guessing, but exclusion may mean respondents who are not confident in their answer will pass the question [32, 33••]. Where there are limited response options, such as in dichotomous questions, effectiveness of the measure may be decreased, as the respondent has a 50% chance of being correct. The use of MCQs with four to five answer options has been suggested as appropriate if there are adequate feasible distracters [32, 33••]. Research comparing MCQs with multiple-answer MCQs (e.g. “Select all statements that are correct”) in a programming course suggested no differences in preference for question type, that multiple-answer MCQs potentially reduced guessing of correct answers, and that this question format could be useful for providing formative feedback [51, 52]. Future questionnaires could consider a mix of MCQ and multiple-answer MCQs from which analysis could be conducted to provide insight into knowledge gaps and misunderstandings of the area assessed.

### Reducing Ambiguous Questions

The assessment of nutrient knowledge was prevalent in reviewed questionnaires. In some questionnaires, this was examined by having the respondent determine whether a food is “high” or “low” in a certain nutrient or by classifying whether a food is a “good source” of a nutrient [23, 24, 26]. This may be ambiguous because what constitutes high or low and/or a good or poor source of a nutrient is not always clear. For example, a question such as “Do these foods contain a high or low content of protein? (Beans/Pulses)” [19] can be difficult to answer, as legumes contain substantially more carbohydrate than protein, so it is “low” compared to meat, but “high” in the context of a vegetarian/vegan diet. A more considered approach may be to provide dietary context as well as moving away from dichotomous answers for minimising these ambiguities.

### The Use of Images to Enhance Readability and Engagement

Only two questionnaires specifically mentioned use of pictures [19, 21]. The use of images, visual elements, and interactive screen design could be leveraged to decrease respondent fatigue and monotony resulting in less incomplete responses and nonresponse bias [53–55]. One questionnaire specifically assessed readability [30]. To ensure an athlete’s knowledge score is not hindered by their level of literacy and to increase accessibility, use of images could reduce ambiguity in interpreting questions and improve comprehension [56, 57]. Similar use of visuals has been used successfully in a general nutrition knowledge instrument; however, this remains largely underutilised in athlete-specific questionnaires [58]. The consideration of visuals in combination with readability tests or audio narration of questions is recommended in the development of future questionnaires.

### Greater Incorporation of Electronic Features

A key benefit of athlete nutrition knowledge assessment is to identify strengths and potential gaps in understanding. Several studies reported that questionnaires were deployed electronically, which represents an opportunity to return timely, automated, and personalised feedback to respondents. However, to date, only one questionnaire has implemented this [42•]. By offering personalised feedback or something that is tangible or meaningful to the user, respondents may answer more truthfully or be more likely to complete [59, 60]. The benefits of feedback in improving learning outcomes are well-established [61], and for athletes can provide a catalyst for self-learning and/or an opportunity to engage with a nutrition professional for assistance [62]. Studies in non-athletes have demonstrated the effectiveness of computer-generated, scored, and personalised feedback in improving diet and lifestyle factors [63, 64]. Feedback is also beneficial for SN in athlete education by providing insight into the effectiveness of and the potential gaps in their education.

### Assessment by Modules

Use of electronic platforms may mean it is feasible to offer a “modular” approach to nutrition knowledge testing. This approach has been reported as a feature that practitioners would like to see in questionnaires [31]. Using this approach, practitioners or researchers administering a questionnaire could select the most relevant topics to suit the needs of the athlete. For example, a module on competition nutrition may be more relevant leading into a season. For a younger group of athletes, a supplements modulemay not be necessary. By developing modules, a questionnaire could be expanded to provide assessment for specific knowledge areas or sport types such as nutrition for travel or for endurance athletes. Establishing the validity and reliability of unique modules would allow each set of questions to be used as a standalone assessment. Further psychometric testing should be conducted to assess whether modules deployed as a series to form a questionnaire remains valid and reliable.

The development of a question bank that provides a rotating or randomly generated set of items that have been psychometrically tested to assess the same construct would further improve the way in which nutrition knowledge is assessed as it is not possible to rule out learning the test on multiple subsequent administrations without truly improving in knowledge. Although test–retest reliability has been used to demonstrate the stability of many questionnaires in this review, the development of a question bank would necessitate parallel-form reliability, where the correlation between original and alternate versions of an instrument is examined [35, 65]. Item response theory techniques have been used in the validation of question banks in other fields [66, 67], which may be relevant given the increase reliance on Rasch techniques identified by this review.

### Modification of Knowledge Questionnaires

The time, resources, and expertise necessary to develop a new tool for a specific population are often impractical. Therefore, it is expected that existing measures will be modified or adapted to suit the population being examined. Where modifications have occurred, it is essential the measure is reassessed for validity and reliability, with the extensiveness of the testing related to the level of modification. A three-level classification system proposed by Coons et al. [68] suggests minor modifications are those that will not change the content or meaning, such as changing font or medium of delivery; moderate modifications are those that include splitting items, altering wording, or item order; and substantial modifications are those that include removing or changing items, their response options, or their wording. Where translation is necessary to reach diverse population groups, the process should be documented. A seven-step framework for the translation, adaptation, and validation of instruments has been proposed by Sousa et al. [69]. Specifically, steps 1–5 may be relevant to researchers looking to adapt questionnaires into their own language, with steps 6 and 7 describing the validation of the translated instrument. More importantly for nutrition-related questionnaires, food items should be aligned culturally and reflect the food supply and terminology used in the country [70]. For example, the relevant usage of prawn/shrimp, soft drink/soda, and the conversion of imperial or metric units should occur when modifying between US and Australian populations. Due to the time-sensitive nature of research, an “in-depth pre-test” conducted on a small sample may be adequate to determine whether modifications were appropriate, and to collect psychometric properties such as item-scale correlations and reliability measures prior to large-scale deployment [71]. It is also suggested that validity tests should match those conducted on the original measure as using different tests may demonstrate the validity, but makes comparison with the original difficult [71]. That said, further testing questionnaire validity outside of what was conducted on the original should not be discouraged.

## Conclusion

Assessment of nutrition knowledge is an important component of providing support to athletes. Nutrition knowledge questionnaires may allow screening of large groups of athletes to prioritise individuals who need advice sooner and evaluate the effectiveness of advice and education initiatives. This review identified 12 new or modified questionnaires published in the last 5 years, with advancements including the use of more sophisticated validation and reliability testing techniques and the use of electronic features to advantage. Areas that need improvement are refinement of question format and using questions that assess procedural (practical application) rather than focussing on declarative (factual) knowledge. Future advancements would include the use of accessibility options for electronic questionnaires, the use of images to enhance readability and engagement, modular structure of questionnaires to provide adaptability, and further development in the provision of electronic feedback.
